# Cutting edge of immune response and immunosuppressants in allogeneic and xenogeneic islet transplantation

**DOI:** 10.3389/fimmu.2024.1455691

**Published:** 2024-09-13

**Authors:** Liting Yue, Jisong Li, Mingjun Yao, Siyuan Song, Xiaoqin Zhang, Yi Wang

**Affiliations:** ^1^ Center of Critical Care Medicine, Sichuan Provincial People’s Hospital, University of Electronic Science and Technology of China, Chengdu, Sichuan, China; ^2^ Department of Gastrointestinal Surgery, Sichuan Provincial People’s Hospital, University of Electronic Science and Technology of China, Chengdu, China; ^3^ Department of Neuroscience, Baylor College of Medicine, Houston, TX, United States; ^4^ Clinical Immunology Translational Medicine Key Laboratory of Sichuan Province, Sichuan Provincial People’s Hospital, Chengdu, China

**Keywords:** islet transplantation, immune response, immunosuppressants, xenotransplantation, allogenic and xenogenic islet transplantation

## Abstract

As an effective treatment for diabetes, islet transplantation has garnered significant attention and research in recent years. However, immune rejection and the toxicity of immunosuppressive drugs remain critical factors influencing the success of islet transplantation. While immunosuppressants are essential in reducing immune rejection reactions and can significantly improve the survival rate of islet transplants, improper use of these drugs can markedly increase mortality rates following transplantation. Additionally, the current availability of islet organ donations fails to meet the demand for organ transplants, making xenotransplantation a crucial method for addressing organ shortages. This review will cover the following three aspects: 1) the immune responses occurring during allogeneic islet transplantation, including three stages: inflammation and IBMIR, allogeneic immune response, and autoimmune recurrence; 2) commonly used immunosuppressants in allogeneic islet transplantation, including calcineurin inhibitors (Cyclosporine A, Tacrolimus), mycophenolate mofetil, glucocorticoids, and Bortezomib; and 3) early and late immune responses in xenogeneic islet transplantation and the immune effects of triple therapy (ECDI-fixed donor spleen cells (ECDI-SP) + anti-CD20 + Sirolimus) on xenotransplantation.

## 1 Introduction

Diabetes is a chronic metabolic disease characterized by high blood glucose levels, affecting over 500 million people worldwide. Type 1 diabetes (T1D) results from an autoimmune response that destroys the insulin-producing β-cells in the body, resulting in the inability to produce insulin to regulate blood glucose levels (1). Since the discovery of insulin in 1922, insulin therapy has been used to treat patients with T1D. This disease requires minute-to-minute regulation of blood glucose levels, and measures such as exogenous insulin supplementation and continuous glucose monitoring (CGM) can have a certain delay in detecting and controlling blood glucose levels, which insulin injections cannot achieve (2, 3). Only by transplanting insulin-producing cells from donors can we precisely measure and deliver the appropriate doses of insulin (4). Additionally, although intensive insulin therapy can improve glycated hemoglobin levels, it does not prevent diabetic complications (5). When patients face severe metabolic complications, failure of exogenous insulin treatment, or when insulin use fails to prevent acute complications, islet transplantation becomes a necessary treatment measure (6). The transplantation of pancreatic tissue, whether whole pancreas or islets, is a clinical option for the treatment of labile type 1 diabetes. Pancreas transplantation is usually performed as a multi-organ transplant procedure; most of these (72%) are combined pancreatorenal procedures. Therefore, it is particularly suitable for patients with type 1 diabetes combined with end-stage renal disease. Open surgery is required to transplant the entire pancreas into the abdominal cavity of the recipient and connect the blood vessels and digestive tract. The operation is complicated and traumatic, and the recovery time is long. Whole organ pancreas transplants restore euglycemia almost immediately following transplantation, and long-term graft survival rates are excellent. Despite the need for immunosuppression, recipient morbidity and mortality decreased significantly, as did the risk of complications associated with poor glycemic control and a better quality of life (7, 8). Islet transplantation refers to the isolation, purification and transplantation of islets from the pancreas of the donor into the recipient (detailed procedures are described below). Islet transplantation is suitable for type 1 diabetes patients who have experienced severe hypoglycemic events. Following Edmonton protocol, the islets are injected directly into the recipient’s liver portal vein under the ultrasound observation, and the operation is less traumatic, the anesthesia time is shorter, the invasion is less, and the recovery time is fast. Although many patients experience significant improvements in blood sugar control after transplantation, exogenous insulin may still be required, and long-term success rates are relatively low.

As an alternative therapy, islet transplantation can sustainably reverse T1D. Successful islet transplantation eliminates the need for stringent blood glucose monitoring and prevents the progression of diabetic complications. However, a significant challenge faced by islet transplantation is the immune response of the body to the foreign islets. When donor islets are exposed to the recipient’s immune system, the implants can trigger a rapid immune response (9, 10). Therefore, the survival rate of islets after isolation and transplantation becomes a major issue. Immunosuppressive therapy is currently the most popular immunomodulation method to ensure the survival of islet grafts. Clinical islet transplantation began in the 1970s (11), but due to various reasons, its clinical efficacy was not ideal. It was not until 1999 that Shapiro et al. (12) proposed and established a standard set, including donor selection, islet equivalent transplantation, and post-operative immunosuppressive regimens. They used a large number of isolated islet cells for transplantation and implemented a new regimen post-operatively, using a steroid-free regimen and reduced doses of calcineurin inhibitors (sirolimus, low-dose tacrolimus, and daclizumab), known as the “Edmonton Protocol” (12). Once this protocol was promoted, clinical results improved significantly, marking an important milestone in clinical IT. With the promotion of the Edmonton clinical protocol and the continuous improvement of islet cell isolation techniques, the survival rate of islet transplantation has significantly improved but is still relatively low compared to other organs. Moreover, it is known that the traditional methods of using immunosuppressive drugs during and after islet transplantation can cause many side effects, such as mouth ulcers, peripheral edema, anemia, weight loss, and paroxysmal diarrhea (9, 13). Therefore, to improve the survival rate after islet transplantation, many issues must be addressed, including islet viability, effective implantation, and the application of immunosuppressants that lead to islet damage (14). Therefore, the purpose of this article is to summarize the immune responses and mechanisms of action of immunosuppressants that occur after islet transplantation to better guide islet transplantation and improve islet survival rates.

## 2 Immune response in allogeneic islet Transplantation

### 2.1 Inflammatory response

Clinical islet transplantation requires four steps: perfusion of the donor pancreas, digestion of the pancreas to separate the islets from the exocrine tissue, purification of the islets, and transplantation via the portal vein infusion of islet into the recipient (15). When the prepared islets are infused into the patient’s body through the portal vein, it triggers an inflammatory response. Early inflammatory response leads to the early loss of islet viability, posing a significant challenge to the long-term survival rate of islet transplantation. This early inflammatory reaction significantly affects islet viability, with estimates indicating that up to 50% of transplanted islets may be lost during this initial phase (16). Post-pancreas transplantation, ischemia-reperfusion creates an inflammatory environment, where the Instant Blood-Mediated Inflammatory Reaction (IBMIR) plays a crucial role. Injecting purified islets into the recipient’s portal vein promotes an innate immune-dependent inflammatory response, known as IBMIR.

**Figure 1:**
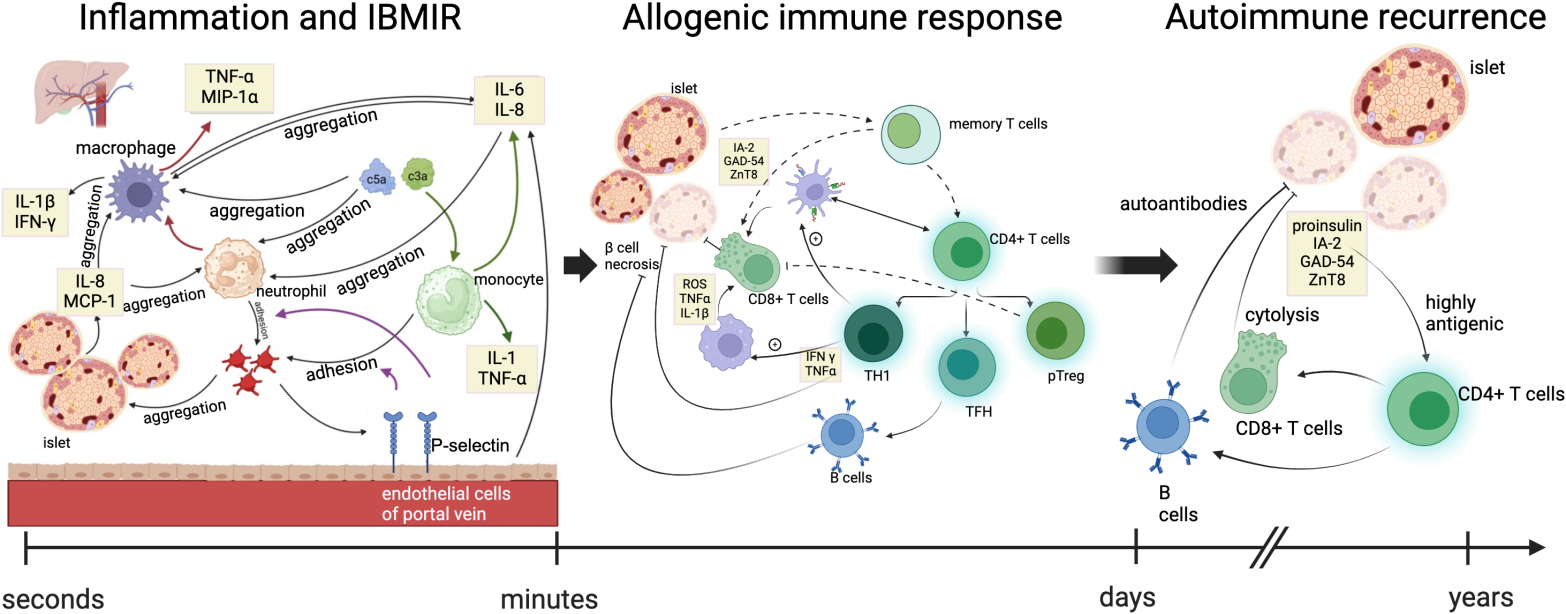
Immune mechanisms at three stages of allogeneic islet transplantation. The immune responses occurring during allogeneic islet transplantation included three stages: inflammation and IBMIR, allogeneic immune response, and autoimmune recurrence. In the early stages of transplantation, islets secrete pro-inflammatory factors and activate the complement system, promoting the recruitment of platelets, neutrophils, and monocyte macrophages to the graft. Vascular endothelial cells secrete cytokines and release P-selectin, promoting the adhesion between monocytes/neutrophils and platelets. Accumulated monocyte macrophages and neutrophils further enhance the recruitment of macrophages and the secretion of cytokines. Green arrows indicate the process by which the complement system promotes cytokine secretion by monocytes. Red arrows indicate the process by which neutrophils enhance cytokine secretion by macrophages. Purple arrows represent the process by which P-selectin promotes the adhesion of monocytes and neutrophils to platelets. Within days after transplantation, the release of inflammatory signals leads to increased cytokine production, with neutrophils signaling macrophages and dendritic cells to the site of islet phagocytosis, presenting antigens on their surface and recruiting adaptive immune cells. The infiltration of helper and cytotoxic T cells further damages the islets, recruiting B cells that produce antibodies against the allogeneic islets and differentiating T cells into memory T cells, ultimately leading to the rejection of the overall allogeneic transplant. TNFα, tumor necrosis factor alpha; MIP1α, macrophage inflammatory protein-1 alpha; IFN-γ, interferon; MCP-1, monocyte chemoattractant protein-1; GAD65, glutamic acid decarboxylase 65; IA-2, insulinoma-associated protein 2; ZnT8, transporter 8. ROS, reactive oxygen species; TH1, T helper 1 cells; TFH, follicular helper T cells; pTreg, peripheral regulatory T cells.

IBMIR is initiated by the intense activation of the coagulation cascade, where the negatively charged surface of the islets activates the intrinsic coagulation pathway (17), and the tissue factor (TF) expressed by the islets induces the extrinsic coagulation pathway (18). Simultaneously, islets secrete inflammatory factors such as IL-8 and MCP-1, which have chemotactic and pro-inflammatory effects on macrophages and neutrophils (19, 20). Activated platelets can adhere by binding to the extracellular matrix (ECM) and collagen on the surface of islets. Additionally, due to the rapid transient expression of p-selectin on the membranes of activated platelet alpha granules and vascular endothelial Weibel-Palade bodies, the p-selectin lectin-like domain present on neutrophils and monocytes binds with sialyl Lewis x and p-selectin glycoprotein ligand-1, mediating the rolling of neutrophils and monocytes on endothelial cells and their adhesion to platelets (21, 22). On the other hand, vascular endothelial cells secrete IL-6 and IL-8, promoting the aggregation of neutrophils and macrophages (19). Complement activation is triggered by natural immune antibodies IgG and IgM. When isolated islets are exposed to blood, the complement system is rapidly activated, leading to the lysis of islet cells. Simultaneously, the production of anaphylatoxins C3a and C5a further induces the aggregation of macrophages and neutrophils, promoting the release of cytokines such as IL-1, IL-6, IL-8, and TNF-a by monocytes (23). Granulocytes appear 8 hours after islet transplantation, with extensive infiltration into the grafts after 12 hours. Neutrophils are the main members of the granulocyte family and the first line of defense in innate immunity. They contain various cytokines that, when activated, are released and cause damage to islets; neutrophils significantly contribute to the activation and recruitment of macrophages at acute inflammation sites. Once activated, they produce various chemokines to attract monocytes and macrophages. Additionally, neutrophil infiltration leads to the release of cytokines such as TNF-a and macrophage inflammatory protein-1a by T cells and macrophages, which can expand IBMIR and induce subsequent adaptive immunity, triggering and enhancing cellular rejection (23, 24) (**Figure 1**). Aggregated macrophages continuously secrete cytokines such as IL-6 and IL-8 to sustain the inflammatory response and release pro-inflammatory factors such as IL-1b, IFN-g, and TNF-a. The IL1b secreted by macrophages and neutrophils binds to IL-1b receptors on the surface of islet cells, activating IL-1 receptor-associated kinases and TNF receptor-associated factor 6, leading to the phosphorylation and degradation of IkB, releasing NF-kB, which then enters the nucleus to regulate the transcription of multiple genes, including IL-1, IL-6, TNF-a, and iNOS. TNF-a produced by macrophages and islet cells binds to TNF receptors, activating the NF-kB and MAPK pathways and inducing apoptosis. Apoptosis is mediated by caspase-3 activation through the MAPK pathway or by activating effector caspases, including FADD-mediated caspase-3 activation. IFN-g produced by macrophages binds to IFN-g receptors on islet cells, activating JAK1 and JAK2. Activated JAK2 then activates Signal Transducer and Activator of Transcription 1 (STAT1). STAT1 is then transferred to the nucleus for gene regulation, ultimately leading to islet cell apoptosis. The pro-apoptotic effect of STAT1 may be partially mediated by the activation of caspase-2, caspase-3, and caspase-7 (25). Under the combined action of cytokines IL-1b, TNF-a, and IFN-g, the overexpression of iNOS in b-cells and macrophages leads to excessive synthesis of NO. Subsequently, NO loses electrons and combines with superoxide radicals to form highly reactive peroxynitrite (ONOO-). The cytotoxicity of ONOO subsequently induces islet cell apoptosis. On the other hand, macrophages play an antigen-presenting role, promoting the activation of T cells into CD8+ T cells and CD4+ T cells. Activated T cells produce cytokines such as IFN-g, TNF-a, and lymphotoxin, thereby inducing b-cell apoptosis. (**Figure 1**). Lisa Özmen et al. (26) exposed human islets to ABO-compatible blood and found that administering Melaglavin dose-dependently eliminated IBMIR. In the absence of or at concentrations below 0.4 μmol/l of Melaglavin, the integrity of islets exposed to blood was lost. However, at concentrations of 1-10 μmol/l, Melaglavin inhibited coagulation and complement activation, leading to reduced platelet and leukocyte activation and consumption. This protective effect indicates that thrombin plays a crucial role in IBMIR and suggests that thrombin inhibition could improve the outcomes of clinical islet transplantation (26). L. Moberg et al. perfused human islets with fresh ABO-compatible blood for 30 minutes. In control samples (containing either only islets or blood with non-inhibitory anti-TF [4503]), coagulation occurred within 15 minutes. However, blood containing inhibitory anti-TF [4509] inhibited coagulation throughout the observation period. The study found that IBMIR is initiated by TF and consistently occurs during clinical islet transplantation, even in the absence of clinical symptoms like portal vein thrombosis. Inhibiting this process may increase the success rate of clinical islet transplantation and reduce the number of donors required per patient (18).

IBMIR, characteristic of innate inflammatory responses and thrombotic pathway, is driven by the activation of the coagulation cascade, with negatively charged islet surfaces activating the intrinsic coagulation pathway, and tissue factor (TF) expressed by the islets triggering the extrinsic pathway.

The innate immune system is the body’s rapid response to an initial infection or injury. In IBMIR, the following components are mainly involved: Neutrophils: They are the first cells to arrive at the transplant site, release inflammatory mediators and oxygen free radicals, mediate local tissue damage and remove pathogens. Monocytes and macrophages: Monocytes are recruited and converted into macrophages, which further release cytokines (such as TNF-α and IL-1) that intensify the inflammatory response and enhance recruitment of immune cells. Cytokines released by neutrophils and macrophages in IBMIR not only promote local inflammatory responses, but may also affect T cell activation and subsequent adaptive immune responses (27).The activation of innate immune cells can lead to apoptosis or necrosis of the transplanted islet cells, thus reducing the survival rate of the grafts. The inflammatory response triggered by IBMIR may cause more immune cells to aggregate, forming positive feedback and further aggravating the damage. There is a close interaction between IBMIR and congenital leukocyte response, which together affect the success rate of islet transplantation.

### 2.2 Allogeneic immune response

The allogeneic immune response, which is adaptive immunity, occurs later but leads to long-term functional reduction of β-cells, resulting in a significant portion of islets losing their insulin independence. Analysis of pancreatic sections from T1D patients reveals significant immune infiltration within individual islets, confirming the crucial role of CD4 and CD8 T cells in β-cell destruction (14, 28). Despite high levels of systemic inflammation markers in T2D patients, their islets do not exhibit similar T cell infiltration, in stark contrast to the pancreatic sections of T1D patients, making islet autoantibodies a differential diagnostic marker between T1D and T2D (4). The presence or development of alloreactivity (against human leukocyte antigens, HLA) and its impact on allogeneic graft survival is well-defined in the solid organ transplantation literature. Donor-specific antibodies (DSA) binding to endothelial cells or islets (which constitutively express Class I HLA and aberrantly upregulate Class II HLA) can activate the classical complement pathway. Even in the absence of complement, some DSAs can promote antibody-dependent cellular cytotoxicity, where innate immune cells bind to Fc fragments, triggering the release of cytolytic enzymes by neutrophils and NK cells. C4d is a degradation product of the classical complement pathway, covalently bonded to the endothelium, serving as a marker for antibody-mediated immunity (4). Transplanting allogeneic islets or pancreas to T1D recipients expressing major and minor histocompatibility antigens on endogenous islets and pancreas can elicit complex adaptive B cell and T cell responses, leading to classical allogeneic graft rejection.

Key effector immune cells include cytotoxic T cells (CD8^+^ T cells), macrophages, plasma cells, and CD4^+^ T helper cells. In human T1D, existing evidence from single-islet studies from the Network for Pancreatic Organ Donors with Diabetes suggests that β-cell destruction is largely mediated by direct contact between CD8 T cells and β-cells, as well as CD4 T cell-mediated M1 macrophage polarization (29–31).

CD8+ T cells eliminate cells presenting non-self antigens by inducing apoptosis through the release of cytotoxic molecules (such as granzymes and perforin) or through cell-surface interactions (such as the binding of Fas ligand (also known as CD95L) on T cells to Fas receptors on the target cells) (32). Activated CD8+ T cells infiltrating the graft also induce macrophage activation, particularly through the expression of pro-inflammatory cytokines such as IFN-γ (33).

Macrophages typically exhibit pro-inflammatory characteristics and display M1 polarization during acute rejection, producing pro-inflammatory cytokines that lead to direct cellular damage and coordinate pro-inflammatory immune responses (34). Their primary function is phagocytosis, recognizing damaged allogeneic graft tissue through pattern recognition receptors such as Toll-like receptors. As antigen-presenting cells, macrophages can present allogeneic antigens on MHC class II molecules, thereby promoting the adaptive immune response (35).

Plasma cells are another type of effector immune cell derived from B cells and form the cornerstone of humoral immunity. They enable the body to combat foreign invaders not only by neutralizing pathogens but also by performing various effector functions, including regulating hypersensitivity reactions, activating the complement cascade, and modulating the mucosal microbiome. However, their activity can be problematic in solid organ transplantation (36). In transplantation, plasma cells can produce donor-specific antibodies (DSAs), which lead to acute and chronic rejection by activating the complement system, resulting in vascular injury and graft loss. The impact of DSAs has been extensively evaluated in various solid organ transplants (37–39).

CD4^+^ T helper cells play a critical role in immune rejection. They coordinate the activation of other immune cells, such as B cells and cytotoxic T cells, to enhance the immune response against allogeneic material. These CD4^+^ T cells are capable of producing and releasing various cytokines, including interferon-gamma (IFN-γ) and interleukin-2 (IL-2). Additionally, CD4^+^ T cells actively interact with B cells, promoting antibody production and thereby strengthening humoral immunity (40, 41). CD4 T cells can provide “help” to B cells and stimulate antibody production (as described above), as well as promote effector CD8 T cell responses and stimulate resident macrophages in the islets (42, 43).

Auto-reactive CD4 T cells interact with dendritic cells presenting islet antigens (44) and can differentiate into T helper 1 (TH1) cells, follicular helper T cells (TFH), peripheral regulatory T cells (pTreg), or anergic cells. TFH cells help B cells produce high-affinity islet-specific antibodies (29). TH1 cells activate dendritic cells and enhance antigen presentation to islet-specific CD8 T cells (45), thereby inducing the proliferation of effector CD8 T cells (45). TH1 cells migrate to the pancreas (46), secrete pro-inflammatory cytokines interferon-γ (IFNγ) and TNFα, and induce β-cell death (47). TH1-derived IFNγ and TNFα stimulate M1 macrophages in the islets to produce reactive oxygen species (ROS), TNFα, and IL-1β (48), further amplifying the cycle of β-cell death (30)The resulting inflammation leads to increased infiltration of CD8 T cells, which directly kill β-cells via perforin and granzyme B (49), while natural and peripheral regulatory T cells (nTreg and pTreg) attempt to suppress this response through TGFβ and IL-10 (50).

### 2.3 Autoimmune recurrence

Patients with T1D and concurrent autoantibodies have a lower success rate for islet transplantation due to the presence of memory CD4+ and CD8+ T cells, which rapidly reactivate to target islet antigens (IA-2, GAD-54, and ZnT8) and destroy the transplanted islets (42, 43). Patients with T1D who have long-term β-cell transplants still have the ability to destroy islets. Reviewing the case of David Sutherland’s identical twin transplant surgery, where the pancreas of an unaffected twin was transplanted into the twin with long-term T1D without immunosuppression, resulted in the loss of transplanted β-cell function and pancreatitis (51, 52). This is because most individuals’ immune systems develop the ability to distinguish self from non-self. In T1D, the loss of the ability to recognize insulin-producing islet β-cells as self leads to an autoimmune response, which destroys β-cells in the natural pancreas (53, 54). This autoimmune response is primarily mediated by T cells, which are the main effector cells in the β-cell destruction process. Moreover, there is ample evidence that isolated allogeneic islet transplants may cause autoimmune recurrence in a small but significant proportion of patients. In the autoimmune process, when islets or pancreas are transplanted into recipients with T1D, donor β-cells express β-cell-specific antigens that are attacked by T cells and B cells (55–58). These include insulin (proinsulin), glutamic acid decarboxylase 65 (GAD65), insulinoma-associated protein 2 (IA2), and zinc transporter 8 (ZnT8), which are highly antigenic to both B cells and T cells in humans (59). This explains why islet autoantibodies sometimes rise sharply within weeks after transplantation. This increase usually occurs without any signs of allogeneic immunity (60). Therefore, transplanting islets or pancreas into T1D recipients is a renewed challenge to the autoreactive memory response and may lead to the recurrence of autoimmune function post-transplant.

In summary, when allogeneic islets are transplanted into T1D patients, a comprehensive immune response is elicited against the foreign tissue. Besides the classic rejection of the allogeneic graft, the outcomes of islet or pancreatic transplantation may be severely impacted by early intense inflammatory responses and the reactivation of autoimmunity. In simple terms, the three stages of immune response experienced are inflammation and IBMIR, allogeneic immune response, and autoimmune recurrence. Within days after transplantation, the release of inflammatory signals leads to increased cytokine production, with neutrophils signaling macrophages and dendritic cells to the site of islet phagocytosis, presenting antigens on their surface and recruiting adaptive immune cells. The infiltration of helper and cytotoxic T cells further damages the islets, recruiting B cells that produce antibodies against the allogeneic islets and differentiating T cells into memory T cells, ultimately leading to the rejection of the overall allogeneic transplant. The entire process of allogeneic transplant rejection may be amplified in T1D patients because they have effectively primed T cells specifically targeting β-cells.

## 3 Immunosuppressants in allogeneic islet transplantation

A major issue in islet transplantation is transplant rejection. To prevent this complication, immunosuppressive drugs such as cyclosporine, tacrolimus, mycophenolate mofetil, and corticosteroids must be used (61). However, immunosuppressants have severe side effects, including inducing diabetes, nephrotoxicity, and carcinogenic effects (62–65).

### 3.1 Calcineurin inhibitors

There are many types of calcineurin inhibitors (CNIs), such as the commonly used cyclosporine and rapamycin. The potent immunosuppressive properties of cyclosporine were discovered in 1976. Cyclosporine blocks the clonal expansion of resting T cells by inhibiting the transcription of genes encoding IL-2 and the high-affinity IL-2 receptor, which is crucial for T cell activation (66).

Tacrolimus (FK506) was the first macrolide antibiotic explored for its effective immunosuppressive properties in 1987 (67, 68). The mechanism of toxicity of tacrolimus is as follows: tacrolimus binds to the immunophilin FK506-binding protein 12 (FKBP12) to form a complex that binds and inhibits the mammalian target of rapamycin (mTOR) kinase, thereby exerting immunosuppressive activity (69, 70). This kinase is a key regulator of cell metabolism, growth, and proliferation. Importantly, inhibition of mTOR by tacrolimus causes cell cycle arrest in the mid-to-late G1 phase, thus potentially inhibiting tumor cell growth and, importantly, its immunosuppressive function by inhibiting T cell and B cell proliferation (71). However, FKBP12 and mTOR are ubiquitously expressed. Therefore, there is a possibility of “off-target” effects on cells other than tumor and immune regulatory cells.

**Figure 2:**
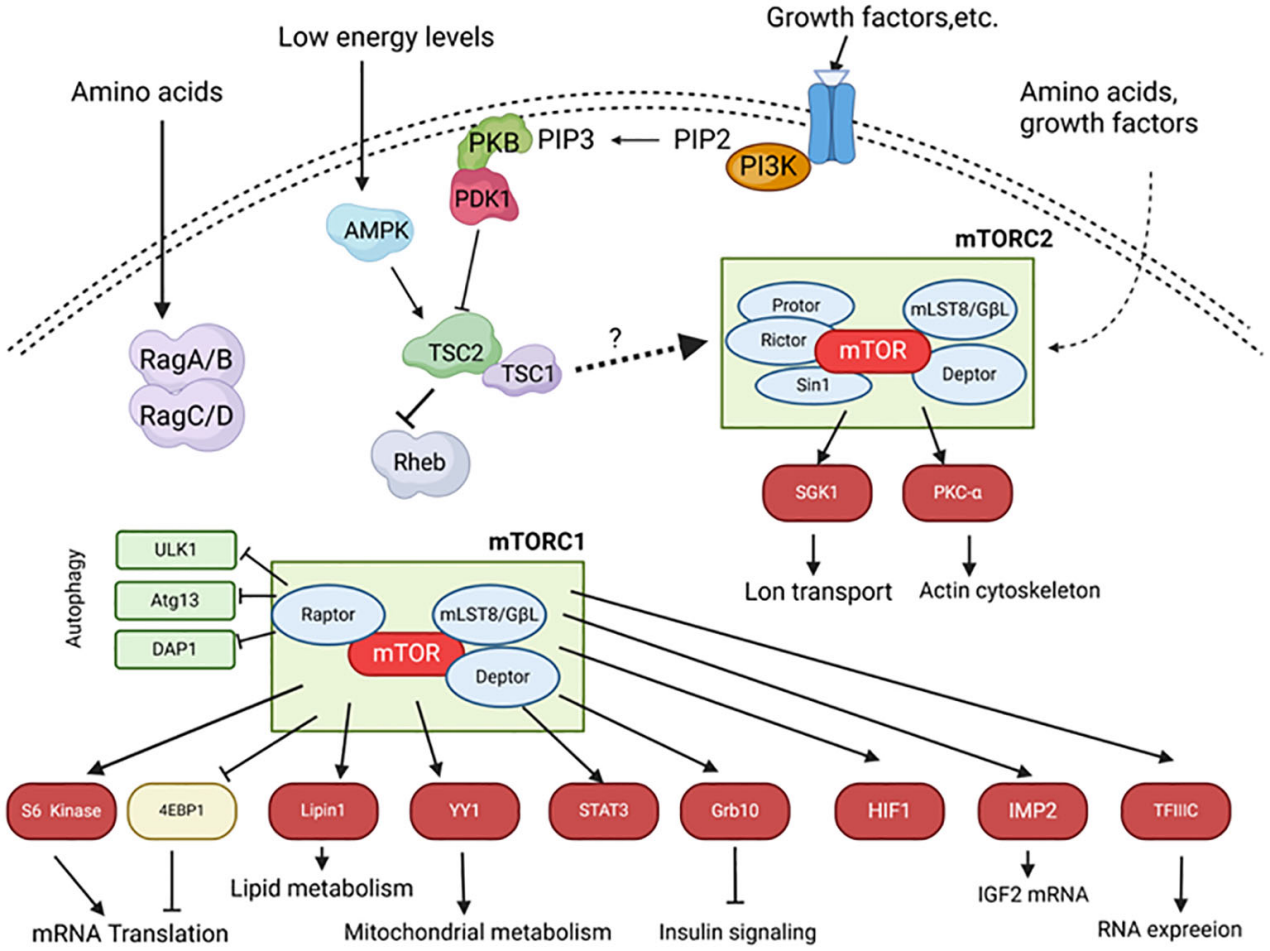
The mTOR signaling pathway in islets. Upon stimulation by insulin and other growth factors, phosphoinositide 3-kinase (PI3K) converts phosphatidylinositol 4,5-bisphosphate (PIP2) into phosphatidylinositol 3,4,5-trisphosphate (PIP3), which localizes PKB to the membrane and activates it through PDK1 and mTORC2. Activated PKB phosphorylates and inhibits TSC1/2. Rheb, a small GTPase inhibited by TSC2, positively regulates mTORC1 activity. mTORC1 phosphorylates S6 kinase 1/2 and 4EBP1, leading to increased mRNA translation. Amino acids activate mTORC1 through Rag A/B and C/D. Under low energy conditions, the ratio of AMP to ATP increases, activating AMP-activated kinase (AMPK), which phosphorylates and activates the TSC1/2 complex, thereby inhibiting mTORC1. mTORC2 activity is primarily mediated through unknown pathways. mTORC2 phosphorylates and activates PKB, serum- and glucocorticoid-induced kinase 1 (SGK1), and PKC. Arrows indicate stimulatory effects; block ends indicate inhibitory effects; solid lines represent direct effects, and dashed lines represent indirect effects. Atg13, Autophagy-related protein 13; DAP1, Death-associated protein 1; Deptor, DEP domain-containing mTOR-interacting protein; 4EBP, eIF4E-binding protein; GbL-G, Protein Gβ-like; HIF1, Hypoxia-inducible factor 1; IMP2, Insulin-like growth factor 2 mRNA-binding protein; mLST8, Mammalian lethal with Sec13 protein 8; PDK1, Phosphoinositide-dependent protein kinase 1; Protor, Protein observed with Rictor; Raptor, Regulatory associated protein of mTOR; Rictor, Rapamycin-insensitive companion of mTOR; Sin1, Stress-activated protein kinase-interacting protein 1; TFIIIC, Transcription factor 3C; ULK1, Unc-51 like kinase 1.

mTOR kinase exists in two distinct complexes: mTOR complex 1 (mTORC1) and mTOR complex 2 (mTORC2). They have different substrates and are regulated differently (**Figure 2**). Although they share some core components, such as mTOR, mLST8, and DEPTOR, they also contain other unique proteins. For example, a unique component of mTORC1 is RAPTOR (regulatory associated protein of mTOR), which acts as a bridge to bind mTOR to its downstream effectors (72, 73). An important component of mTORC2 is the protein Rictor (rapamycin-insensitive companion of mTOR), which is necessary for the formation of the mTORC2 complex and its kinase activity (74, 75). Importantly, mTORC1 is highly sensitive to inhibition by rapamycin, whereas mTORC2 was initially thought to be resistant to rapamycin (74, 75), but in fact, it is sensitive to long-term rapamycin treatment in some cell types (76–78). Therefore, both complexes may play a role in the immunosuppressive and toxic effects of rapamycin. Consistent with its role as a key regulator of cell metabolism, proliferation, and growth, mTORC1 activity is regulated by nutrients, growth factors, and cellular energy levels (**Figure 2**). The best-characterized targets of mTORC1 are eIF4E-binding protein (4E-BP) and S6 kinase protein (S6K), both of which play important roles in the regulation of protein synthesis.

The role of mTORC1 in B cell function is as follows. An important aspect of maintaining glucose homeostasis is the maintenance of pancreatic B cell mass and the ability of B cell mass to increase in insulin-resistant states such as obesity. The increase in B cell mass is due to increased neogenesis (progenitor cell generation) and proliferation (hyperplasia), hypertrophy, and reduced apoptosis. There is substantial evidence indicating that rapamycin significantly reduces the proliferation of B cells and progenitor cells, thereby affecting the maintenance of B cell mass. The most compelling evidence for the role of mTORC1 in regulating B cell mass comes from *in vivo* transgenic mouse models (79). Overactivation of mTORC1 by selectively overexpressing Rheb (80) or deleting TSC1 (81) or TSC2 (81, 82) in B cells leads to increased B cell size and mass, along with improved insulin secretion and glucose tolerance. These effects may be partially mediated by S6K, as mice lacking S6K1 or rpS6 exhibit hypoinsulinemia and glucose intolerance with reduced B cell size (83, 84). Additionally, transgenic mice overexpressing constitutively active S6K exhibit improved glucose tolerance and enhanced insulin secretion with increased B cell size (85). Although these studies strongly suggest the critical role of mTORC1, manipulation of mTOR upstream regulators (e.g., Rheb) may affect pathways beyond mTORC1, so causality cannot be definitively established. There is extensive work investigating the role of mTOR in regulating cell proliferation in certain cell types, but little is known about the exact mechanisms by which mTORC1 signaling regulates B cell cycle progression. However, it is known that mTORC1 can regulate the synthesis and stability of cyclins D2 and D3 in B cells (86). These cyclins form complexes with cyclin-dependent kinase 4, controlling cell cycle progression. In rat islets treated with rapamycin, reduced levels of cyclins D1 and D2 were observed, accompanied by decreased β-cell proliferation (87).

mTORC1 also appears to play a role in insulin secretion by pancreatic B cells. Knockdown of TSC1 in mice results in significantly increased insulin production, independent of B cell mass (81). Additionally, long-term rapamycin treatment inhibits glucose-stimulated insulin secretion (GSIS) in cloned B cell lines as well as rodent and human islets. However, it is unclear whether this effect is mediated by mTORC1 or mTORC2. The control of insulin secretion in B cells involves many complex signaling pathways, and the mechanism by which rapamycin regulates insulin secretion remains unknown. One proposed mechanism is that inhibition of mTORC1 reduces mitochondrial function, particularly the activity of α-ketoglutarate dehydrogenase. This leads to reduced carbohydrate metabolism, thereby decreasing mitochondrial ATP production (88), which is known to regulate insulin secretion in B cells (89). Another explanation is that rapamycin promotes autophagy, a process primarily controlled by mTORC1 rather than mTORC2, or intracellular degradation of cytoplasmic proteins involved in insulin production, leading to inhibition of insulin secretion (89).

It is not completely clear how the activity of mTORC2 is regulated, but there is evidence that it can be stimulated by amino acids and growth factors (90, 91). Downstream targets of mTORC2 include protein kinase C (PKC)-α (85–87) and protein kinase B (PKB) (92), two serine/threonine kinases that play roles in the regulation of key cellular processes such as apoptosis, proliferation, motility, and differentiation, as well as serum- and glucocorticoid-induced kinase 1 (93), which plays a role in the control of ion transport (94) (**Figure 2**).

In mice, B cell-specific deletion of the Rictor gene (an important component of mTORC2) is associated with reduced plasma insulin levels due to decreased insulin secretion from islets, leading to hyperglycemia (95). This is related to reduced B cell mass and proliferation but does not increase B cell apoptosis. Research by Adam D. Barlow et al. has demonstrated that knocking down Rictor in rat islets using small interfering RNA results in increased B cell apoptosis and reduced GSIS (76). These studies specifically demonstrate that mTORC2 activity plays a dominant role in B cell survival and function. Importantly, prolonged rapamycin treatment (24 hours) of MIN6 cells, rat islets, or human islets leads to dissociation of mTORC2, thereby inhibiting its expression. This precedes the toxic effects of rapamycin on function and activity, occurring simultaneously with reduced PKB phosphorylation and downstream signaling. Interestingly, the expression of constitutively active PKB in MIN6 cells and rat islets can mitigate the harmful effects of rapamycin on GSIS and cell viability (76). Overall, this suggests that rapamycin B cell toxicity is primarily mediated through inhibition of mTORC2 and its subsequent impact on PKB signaling. However, this is based on *in vitro* experiments with B cells and needs to be further confirmed *in vivo*.

Extensive research indicates that PKB, as a key downstream effector of mTORC2, plays an important role in B cell survival and function. These studies further reveal the potential role of mTORC2 in B cell homeostasis. For instance, transgenic mice expressing constitutively active PKB in B cells show a significant increase in B cell mass due to increased B cell number and size (96, 97). This is manifested by significantly elevated plasma insulin levels, improved glucose tolerance, and resistance to streptozotocin-induced diabetes. In INS-1 cells, rat B cell lines, and primary rat B cells, expression of constitutively active PKB has also been shown to protect against lipotoxicity (98), cytokine-induced cytotoxicity (99), and AMPK-mediated cytotoxicity (100). Conversely, studies in transgenic mice lacking PKB show significantly elevated blood glucose levels, reduced insulin levels, and impaired glucose tolerance.

Rapamycin is a key immunosuppressant, particularly in islet cell and kidney transplantation. However, extensive *in vitro* and *in vivo* evidence strongly suggests that rapamycin has harmful effects on pancreatic B cells and peripheral insulin sensitivity. This toxicity is mainly because rapamycin inhibits mTOR, which is part of complex signaling pathways controlling many important cellular functions (including mRNA translation, cell proliferation, cell growth, differentiation, protein synthesis, angiogenesis, and apoptosis) through mTORC1 and mTORC2 (71). In summary, rapamycin-induced B cell toxicity and insulin resistance are likely mediated primarily through mTORC2 rather than mTORC1 (76, 95).

In addition to the above mechanisms, although rapamycin is structurally unrelated to cyclosporine, it shares many intracellular pathways that inhibit calcineurin and subsequently block IL-2 production. It acts by limiting the dephosphorylation and translocation of nuclear factor of activated T cells (NFAT). NFATs play a critical role in T cell activation. When T cells are stimulated by antigens, intracellular calcium levels rise rapidly, activating calcineurin. Activated calcineurin dephosphorylates NFATs, exposing nuclear localization signals and causing NFATs to translocate from the cytoplasm to the nucleus. In the nucleus, NFATs bind to specific DNA sequences, regulating the transcription of related genes and participating in T cell proliferation, differentiation, and cytokine production. Calcineurin signaling is essential for insulin secretion and β-cell proliferation (101), and specific inactivation of calcineurin in β-cells is associated with age-related hyperglycemia (102). Apoptosis of islet cells related to calcineurin inhibition is also thought to occur through the limitation of cAMP response element-binding protein (CREB), which reduces the expression of insulin receptor substrate-2 (IRS-2), limits Akt phosphorylation, and affects insulin secretion (103, 104). CNIs also reduce the expression of cell surface glucose transporter 4 (GLUT4) and decrease insulin-stimulated glucose uptake in adipocytes (105), potentially leading to peripheral insulin resistance.

Moreover, rapamycin promotes reduced mitochondrial Ca2+ uptake, which has been shown to impair respiration and ATP production, leading to impaired glucose-stimulated insulin secretion (GSIS) (106). CNIs, particularly rapamycin, enhance the deleterious effects of glucolipotoxicity on β-cells, inducing the expression of forkhead box protein O1 (FoxO1), thereby limiting proliferation (107), and reducing insulin content and secretion (108). Rapamycin causes reversible graft dysfunction, characterized by amyloid deposition and macrophage infiltration in transplanted islets (101), with no clear evidence of β-cell death. Ultrastructural examination of the grafts shows reduced insulin granules, accompanied by increased transcripts associated with extracellular matrix deposition and inflammation. Heparin is primarily used to reduce IBMIR-mediated cell destruction of islets, promoting the fibrillation of human islet amyloid polypeptide (IAPP) and has been shown to simultaneously promote amyloid deposition and reduce β-cell apoptosis (109). Rapamycin exerts its antifibrotic function by inhibiting JAK2/STAT3 signaling activation through targeting JAK2, thereby inhibiting M2 macrophage polarization (110). After transplanting human islets into NSG mice, rapamycin inhibits β-cell function by activating islet-resident macrophages through inhibition of the NFAT pathway and by stimulating macrophages to produce IL-1β through increased amyloid deposition in the transplanted islets (111). Heparinase treatment significantly reduces amyloid deposition and subsequent β-cell toxicity (112).

As two types of CNIs, cyclosporine and rapamycin have similar mechanisms of action, but rapamycin has been shown to be 10-100 times more potent than cyclosporine in inhibiting mixed lymphocyte cultures and the generation of cytotoxic T cells *in vitro* (66).

### 3.2 Mycophenolate Mofetil

In 1993, Mycophenolate Mofetil (MMF), the 2-4 morpholinoethyl ester of the biologically active compound mycophenolic acid, was introduced as a new immunosuppressant (113). MMF reversibly inhibits inosine monophosphate dehydrogenase (IMPDH), a key enzyme in the *de novo* synthesis of the purine nucleotides in DNA (i.e., guanine and adenine) (114). Lymphocytes play a crucial role in graft rejection, and without IMPDH, they cannot produce sufficient amounts of purines (115). Consequently, MMF can prevent the proliferation of T cells and B cells, thereby inhibiting antibody production. Additionally, by lowering intracellular GTP levels in lymphocytes, MMF inhibits glycosylation and the expression of certain adhesion molecules, thus reducing lymphocyte migration to the graft (116). However, its effect on T cell proliferation has garnered more attention due to the critical role of T cells in the allogeneic response (117). MMF is considered a safe drug, with the most commonly reported side effects being mild and primarily involving the gastrointestinal system (diarrhea, abdominal pain, nausea, and vomiting) (118–120). Its main advantage is the lack of nephrotoxicity and diabetogenic effects, making MMF an important drug in kidney and islet transplantation.

### 3.3 Glucocorticoids

T1D is secondary to the initial autoimmunity of islets, resulting from the inflammatory destruction of β-cells (74). Inflammatory macrophages are key in maintaining islet injury (121). Pro-inflammatory cytokines, partly derived from macrophages and damaged β-cells, further inhibit β-cell function by inducing nitric oxide production (122, 123). As T1D progresses, pro-inflammatory cytokines inhibit β-cell regeneration, stimulate peripheral insulin resistance, and maintain insulin inflammation (124). Glucocorticoids (GC) are used clinically for their powerful anti-inflammatory and immunosuppressive effects (125), but high doses of glucocorticoids promote peripheral insulin resistance and inhibit β-cell function (62, 63, 126), thus discouraging their use in T1D treatment and transplantation protocols (12). However, the general notion that GC’s effects on β-cells are purely harmful has been increasingly challenged (127–131). It has now been demonstrated that selective GC regeneration within β-cells can prevent inflammatory β-cell destruction, suggesting that GC-targeting therapy with 11β-hydroxysteroid dehydrogenase type 1 (11β-HSD1) may improve the course of T1D and islet transplantation aggravated by high-dose hormones.

### 3.4 Bortezomib

In addition to the health risks posed by infections and cancer due to broad immunosuppression, numerous studies report that widely used immunosuppressants such as glucocorticoids or calcineurin inhibitors are cytotoxic to islet β-cells (71, 132). Thus, there has been a need to develop tolerance-promoting regimens that can retain the viability and function of islets post-transplantation. Bortezomib, a selective inhibitor of the 26S proteasome, has been FDA-approved for treating relapsed multiple myeloma (133, 134). Bortezomib’s mechanism of action involves inhibiting the proteasomal degradation of IκB, thereby inhibiting the activation of nuclear factor κB (NF-κB) (135, 136). Since NF-κB is a key transcription factor involved in the expression of various genes related to immune responses, numerous studies have demonstrated the immunosuppressive effects of bortezomib. It selectively depletes alloreactive T cells *in vitro* and reduces the secretion of T helper 1 (Th1) cell cytokines (137). Additionally, bortezomib can modulate the function of dendritic cells (DCs): treatment with bortezomib induces a skewed phenotypic maturation of DCs in response to lipopolysaccharides (LPS) and other endogenous stimuli while reducing cytokine production (138). Other studies have also reported that bortezomib can prevent graft-versus-host disease (GVHD) and allograft rejection in mouse models of allogeneic stem cell and cardiac transplantation (139, 140). Furthermore, bortezomib can inhibit the activation of rapamycin-resistant memory T cells without affecting the viability of regulatory T cells (Tregs) in non-human primates (141). Overall, these immunomodulatory effects suggest that bortezomib has the potential to be a promising immunosuppressant candidate in islet transplantation.

So-Hee Hong et al. (142) conducted a related study using BALB/c spleen cells to pre-sensitize C57 BL/6 mice, administering low-dose bortezomib (0.1 mg/kg) for 4 consecutive days to observe its immunosuppressive effects *in vivo*. Since NF-κB is the primary transcription factor for DC maturation, DC maturation status was detected by measuring the expression levels of MHC class II molecules and other co-stimulatory molecules in CD11c+ DCs. The conclusions suggested that low-dose bortezomib only reduced the expression of MHC class II molecules without affecting other co-stimulatory molecules expressed on DCs. Unlike other studies showing bortezomib’s inhibitory effect on alloreactive T cells with high-dose treatment, short-term low-dose bortezomib treatment did not significantly affect the percentage of splenic effector memory cells (CD4+CD44 and CD8+CD44) and the number of T cells producing allogeneic antigen-specific interferon-γ. Based on these results, it was speculated that low-dose bortezomib might inhibit DCs *in vivo* by altering their MHC class II expression.

Additionally, some studies suggest that high-dose rapamycin treatment impairs β-cell regeneration and reduces islet engraftment, adversely affecting islet transplantation (143, 144). Therefore, So-Hee Hong et al. (142) developed a new combination therapy based on low-dose bortezomib and rapamycin, which is highly tolerable and minimally cytotoxic to β-cells, as a potential alternative and tolerance-promoting immunosuppressive regimen in allogeneic islet transplantation. They tested the efficacy of low-dose bortezomib alone or in combination with rapamycin in an islet transplantation model. Low-dose (0.1 mg/kg) bortezomib treatment groups showed longer graft survival rates compared to control groups (0.05 mg/kg group: P=0.1, 0.1 mg/kg group: P=0.0036). Low-dose (1 mg/kg) rapamycin was added to the same transplantation environment. Compared to the control group, the 0.05 mg/kg bortezomib + rapamycin group (P=0.0011) and the 0.1 mg/kg bortezomib + rapamycin group showed significantly prolonged islet graft survival (P=0.001). Although not statistically significant, the combination of rapamycin and bortezomib increased graft survival compared to the bortezomib-only treatment group. In the 0.1 mg/kg bortezomib plus rapamycin treatment group, 4 out of 6 mice maintained normoglycemia for 100 days, while 2 out of 6 mice in the 0.1 mg/kg bortezomib-only treatment group maintained normoglycemia for 100 days. Additionally, the mean graft survival period increased from 24 days to 58 days after adding rapamycin to the 0.05 mg/kg bortezomib treatment group. To determine whether low-dose bortezomib + rapamycin treatment induces immune tolerance, grafts were removed from recipient mice that maintained normoglycemia for over 100 days, and a second graft (islets from BALB/c donors) was transplanted into the contralateral kidney. Interestingly, mice with the second graft maintained normoglycemia for 50 days without any immunosuppression. To determine whether this tolerance was systemic, BALB/c and C3H (third-party) as well as C57 BL/6 (control) skin grafts were transplanted into the flank of the second transplant recipients. The C3H skin grafts were rejected on day 14 post-transplant (DPT). The rejection of BALB/c skin grafts was somewhat delayed but ultimately rejected on DPT 18. Unexpectedly, the rejection of BALB/c skin appeared to result in the rejection of the second islet graft, as blood glucose levels returned to hyperglycemia 20 days after the skin rejection reaction. Thus, it was concluded that this combination therapy induced tolerance to islet-specific antigens, and its inhibitory effect was insufficient to prevent strong skin graft rejection. Many studies have shown that Th1 cells are major participants in graft rejection in various transplant models, with interferon-γ playing a key role by activating cytotoxic CD8+ T cells (145, 146). So-Hee Hong et al. investigated whether bortezomib alone or in combination with rapamycin could reduce Th1 and interferon-γ-producing cells. Splenocytes from allogeneic islet transplant mice that maintained normoglycemia for over 60 days were stimulated *in vitro* with irradiated BALB/c splenocytes, followed by ELISPOT analysis. The combination therapy group showed almost no detectable interferon-γ-producing cells. Although a reduction in interferon-γ-producing cells was also observed in the bortezomib-only group, it was not as pronounced as in the combination therapy group. Moreover, no significant changes were observed in other cytokine-producing cells in the combination therapy group. MLR assays were used to detect BALB/c-specific T cell responses in recipient mice treated with bortezomib + rapamycin. The results indicated that the mice’s T cells had a lower proliferative response to BALB/c antigens but not to third-party C3H antigens. Therefore, these results suggest that low-dose, short-term combination therapy with bortezomib and rapamycin significantly increases graft survival and induces tolerance to islet antigens while inducing severe BALB/c-specific T cell hyporesponsiveness, increased Tregs, and reduced inflammatory cytokines (142).

## 4 Xenotransplantation

The increasing number of patients in need of organ transplants has made xenotransplantation of islets a potential future treatment option for diabetic patients due to the shortage of organ donors. According to recent advances in preclinical studies on non-human primates, porcine islets may be the ideal choice among various animal organs and tissues for xenotransplantation (147), mainly due to the biochemical compatibility of porcine and human insulin and the potential to obtain a large number of donor pigs through relatively short turnover breeding strategies. Additionally, another theoretical advantage of porcine islets is their potential resistance to autoimmune recurrence against human β-cells (148). The main barrier to interspecies transplantation is the preformed xenogeneic antibodies that cause hyperacute rejection. Hyperacute rejection (HAR) is a rapidly occurring rejection in islet transplantation and other organ transplants, usually occurring within minutes to hours after transplantation. This condition arises from the interaction between pre-existing antibodies from humans or non-human primates (NHP) and the antigens present in the graft (149).Among these antibodies, the most common are IgMs and IgGs that identify galactose-α1,3-galactose (α-Gal) residues, which are attached to glycoproteins and glycolipids by the α1,3 galactosyltransferase (α1,3GT) found in non-primate genomes. Humans and apes do not have α-Gal epitopes (150). Furthermore, approximately 70–90% of these antibodies specifically target α-Gal epitopes (151). As a result, when an organ from a pig is transplanted into a human or a non-human primate (NHP), the existing anti-Gal antibodies attach to the α-Gal epitopes found on the graft’s vascular endothelium. This interaction triggers the production of complement component 3b (C3b), activates the complement system (152), and leads to the formation of a membrane attacking complex (MAC).These responses result in the lysis of endothelial cells, damage to the vasculature, and ultimately, rejection of the graft (153, 154). Additionally, the disruption of endothelial vascular integrity leads to interstitial hemorrhage, tissue ischemia, and necrosis (155, 156). Additionally, the failure of the graft is exacerbated by thrombotic occlusion of capillaries, fibrinoid necrosis in arterial walls, and the accumulation of neutrophils (157). The histopathological characteristics of hyperacute rejection (HAR) include compromised vascular integrity, edema, thrombi rich in fibrin and platelets, as well as interstitial hemorrhage accompanied by extensive deposition of immunoglobulins and terminal complement products on the walls of vessels (157). In order to reduce the occurrence of hyperacute rejection, the following measures can be taken: first, immunosuppressants mentioned in this paper are the main measures; knocking out the a1,3GT gene in pigs (GTKO pigs) (158).With the identification of carbohydrate xenoantigens (159) and advances in genetic engineering, it is possible to eliminate these xenoantigens (160) to prevent hyperacute rejection. However, T-cell-mediated xenogeneic immune responses are very intense and more challenging to control compared to immune responses against allogeneic antigens (161).

The xenogeneic T-cell response to porcine islets can be triggered through both direct and indirect antigen presentation (162). Once activated, T cells can mediate graft destruction through direct cytotoxicity (163) or by differentiating into cytokine-producing helper T cells that assist B cells in class switching and antibody production, or by activating innate cells such as macrophages and NK cells involved in xenotransplant rejection (164, 165). Th1 and Th2 cytokines, such as IFN-γ and IL-4, play significant roles in this process (166–168). It has been experimentally demonstrated that the infusion of carbodiimide-fixed donor splenocytes (ECDI-SP) can exert effective immunoregulatory effects through the silent clearance of apoptotic cells, effectively inducing donor-specific tolerance (169–172).

### 4.1 Early acute inflammatory response

Islet xenotransplantation represents a promising therapeutic alternative for treating type 1 diabetes. However, shortly after transplanting donor islets into the recipient, a robust innate immune response is triggered, including an IBMIR, which adversely affects the functionality of the islet transplant (153).

**Figure 3:**
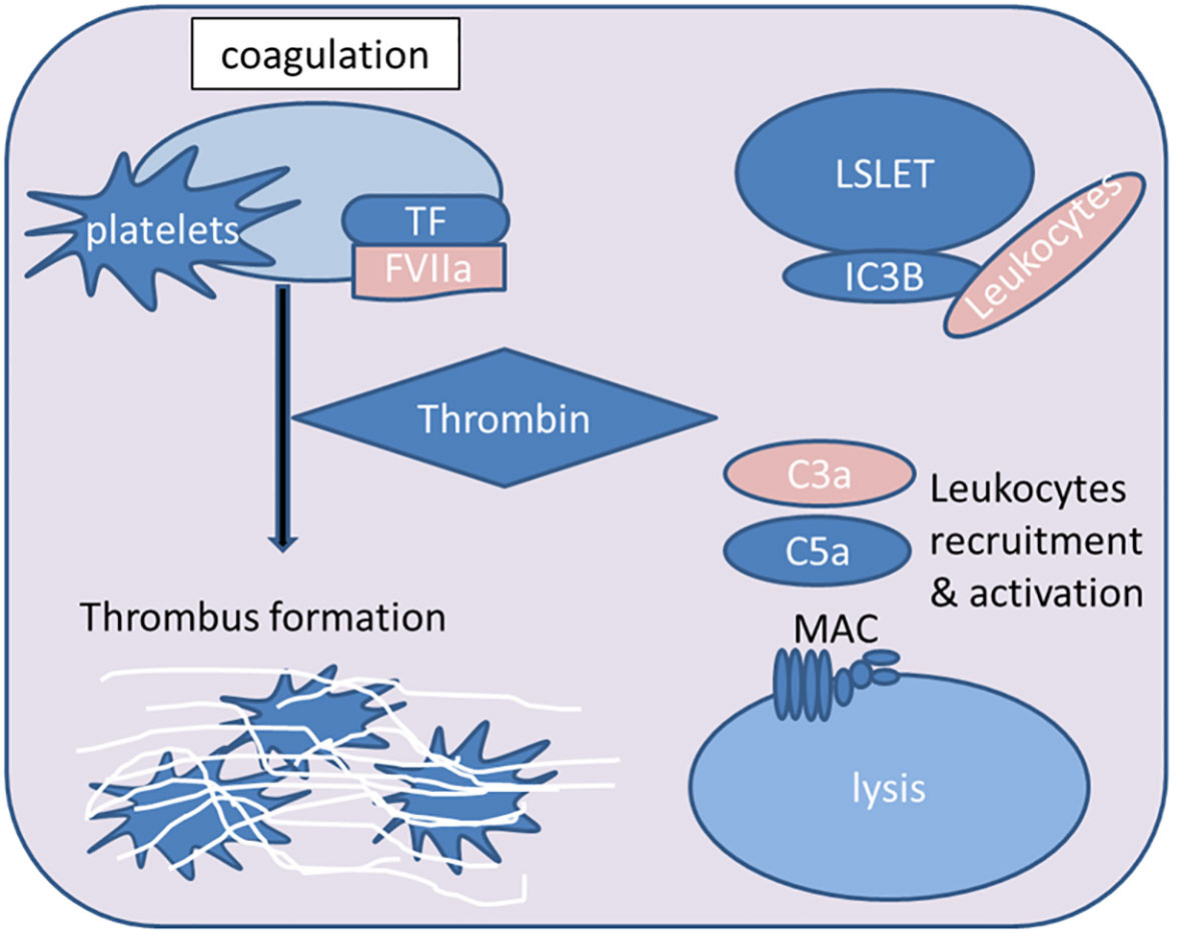
Overview of key steps in the IBMIR process during islet xenotransplantation. The contact between xenogeneic blood and islets triggers the activation of the extrinsic coagulation pathway mediated by tissue factor (TF). Consequently, downstream effector thrombin is produced, leading to fibrin deposition and thrombosis. The attachment of platelets to the islets further supports the pro-coagulant effect. Activated complement fragments (iC3b) deposit on the islet surface, and the anaphylatoxins C3a and C5a activate and attract leukocytes. The formation of the membrane attack complex (MAC) mediates islet lysis (FVIIa, activated coagulation factor VII; MAC, membrane attack complex).

IBMIR is triggered by the xenogeneic contact between blood and islets, involving the activation of coagulation and complement systems, as well as complex interactions between leukocytes and platelets, which significantly impact the function and survival of xenografts, thereby adversely affecting the outcomes of islet xenotransplantation (17, 173) (**Figure 3**). Therefore, the following section explains the mechanisms of IBMIR components.

Research for IBMIR found that platelet-independent complement activation was observed 30 minutes after porcine islets were exposed to plasma, and the formation of membrane attack complexes could be observed in porcine islet tissue pathology sections 60 minutes later, with up to 40% of islets losing their function (174). Complement system activation occurs through three different pathways (known as the classical pathway, lectin pathway, and alternative pathway), depending on the nature of the initial trigger. Regardless of the activation pathway, all pathways converge at the cleavage of C3 by C3 convertase. C3 convertase cleaves the central component C3 into the anaphylatoxins C3a and C3b (175), with the primary function of C3b and its cleavage product iC3b being opsonization for phagocytosis. Additionally, iC3b can bind to complement receptors CR3 and CR4, leading to immune cell adhesion and activation (176, 177). Since complement activation is associated with the proteolytic cleavage of its components, proteases represent another “non-traditional” pathway of complement activation (178, 179).

The classical pathway (CP) is triggered by antigen-antibody complexes recognized by C1q. A major process in this pathway is the production of CP C3 convertase C4b2b, generated by the cleavage of C4 into C4a and C4b, followed by the splitting of C2 into C2a and C2b (180). Activation of the lectin pathway (LP) is initiated by the binding of mannose-binding lectin (MBL) or ficolins to pathogen surfaces, involving the participation of MBL-associated serine proteases MASP-1 and MASP-2, which is significantly similar to CP activation (181).

The spontaneous hydrolysis of C3 to C3(H2O) accounts for the constitutive and continuous low-level activation of the alternative pathway (AP) (182). The generated C3b assembles the APC3 convertase C3bBb together with factor B and factor D (183). The APC3 convertase complex is stabilized by the binding of properdin (184–186).

In all three pathways, the cleavage of C3 to produce C3b is a major component of C5 convertase, which cleaves C5 into the anaphylatoxins C5a and C5b (187). C5b participates in the formation of the membrane attack complex (MAC) by recruiting complement components C6, C7, C8, and C9, with the primary function of mediating the lysis of pathogens or target cells (188).

On the other hand, C3a and C5a anaphylatoxins, by interacting with G-protein-coupled C3a and C5a receptors, are highly effective chemoattractants, promoting the recruitment of inflammatory cells to sites of injury or infection. Furthermore, C3a and C5a can activate immune cells, upregulating the expression and release of inflammatory cytokines and mediators (175, 189).

The coagulation cascade is involved in both hemostasis and thrombosis (190). The tissue factor of the so-called extrinsic pathway is a core participant in coagulation (191), involved in the pathology of thrombosis, including cardiovascular diseases (192, 193) and biomaterial-related processes (194). Inflammatory stimuli or endothelial cell activation produce the extrinsic factor X complex composed of TF and activated coagulation factor VII (FVIIa) (195). The extrinsic factor X complex, in turn, promotes the activation of factor X (FX), which, together with activated FVa and Ca2+, forms the prothrombinase complex that mediates the conversion of prothrombin to thrombin (196). Thrombin can activate platelets, cleave prothrombin into thrombin, leading to the formation of insoluble thrombin (197).

Coagulation and thrombosis are involved in acute reactions to both allogeneic and xenogeneic islet transplantation (18) (198). Notably, exposure of human or porcine islets to human blood results in rapid activation of coagulation, evidenced by upregulated TF levels (199) and significant thrombin generation (26). Moreover, allogeneic islet transplantation is associated with thrombotic manifestations, such as fibrin deposition and the localization of transplanted islets within thrombi (198). Therefore, endogenous antithrombotic agents are significant as potential beneficial modulators of IBMIR. The fine-tuning of the coagulation cascade (200) is mediated by antithrombin III (ATIII), which inactivates thrombin, FXa, and FIXa (201); activated protein C (APC), which, along with protein S, blocks FVa and FVIIIa (202); tissue factor pathway inhibitor (TFPI); and thrombomodulin (TM). TFPI binds and inhibits FXa or the TF/FVIIa complex (203). TM’s anticoagulant activity is mediated by binding to thrombin. The TM-thrombin complex further promotes the generation of APC (204). However, thrombin bound to TM can cleave and activate thrombin-activatable fibrinolysis inhibitor (TAFI) (205), conferring procoagulant properties by blocking fibrinolysis. In the context of xenogeneic islet transplantation, transgenic pigs overexpressing hemostasis-regulating molecules have been generated. For this purpose, the expression of hTFPI protected the xenografts, promoting the achievement of normoglycemia after xenotransplantation. Porcine TM has been shown to be a poor cofactor for human thrombin, resulting in the loss of its protective function and increased coagulation (206). Thus, transgenic overexpression of hTM in pigs can avoid the thrombotic manifestations observed after xenotransplantation of porcine islets (207).

The contact of host blood with transplanted islets rapidly triggers a series of thrombo-inflammatory responses, including upregulation of TF expression (199) and thrombin generation (26). Additionally, the induction of TAFI further propagates the procoagulant effect (208). Intravascular coagulation is induced (209), forming thrombi that capture the islets (198). Concurrently, activation of CP and AP of the complement system occurs, generating anaphylatoxins that lead to the recruitment of inflammatory cells to the graft. Moreover, active complement fragments deposit on the graft, promoting complement-dependent islet lysis (210). Platelets and leukocytes infiltrate the transplantation site and adhere to the islet surface (26, 211). Consequently, the integrity of the islet grafts is compromised, leading to substantial early loss of transplanted islets (212, 213). The acute destruction of a significant proportion of transplanted islets by IBMIR is the primary reason why a high number of islets are required for effective islet transplantation (214). Interestingly, the degree of islet damage increases with the decreasing compatibility between donor and recipient species. Therefore, in the case of xenogeneic islet transplantation, IBMIR becomes more relevant because the recipient cannot control IBMIR induced by xenogeneic islet transplantation due to incompatibility between regulators and effectors, respectively, for the IBMIR of xenografts and recipient cells (215). Furthermore, regulatory proteins are considerably lacking in porcine islet preparations (216). Thus, developing effective treatment regimens targeting the regulatory parameters of IBMIR is imperative (173) (**Figure 3**).

In further studies targeting IBMIR, Bennet et al. cultured isolated islets in whole blood in the presence of soluble CR1 (sCR1). They demonstrated that sCR1 treatment blocked complement activation associated with IBMIR and protected the islets from damage. Simultaneous inhibition with sCR1 and heparin eliminated the adverse effects of IBMIR by reducing the activation of coagulation, complement, and leukocytes. Interestingly, *in vivo* experiments confirmed the protective effect of sCR1, as the use of this inhibitor supported islet integrity, which could be evaluated by the reduction in insulin release shortly after transplantation (198).

Notably, isolated islets can act as a source of procoagulant factors. TF, the main trigger of coagulation *in vivo*, has been found in isolated islets (18, 199), and its knockout (217, 218) or inhibition with specific antibodies (219) has been shown to be beneficial in blocking IBMIR. Interestingly, nicotinamide (a vitamin B derivative) has been used to reduce the expression levels of TF and coagulation, thereby improving IBMIR (20), and leading to improved islet function after transplantation (220).

Islet xenografts can be considered as foreign biological surfaces, and exposure to recipient blood triggers a strong innate immune response. Therefore, an emerging strategy to eliminate the adverse effects of IBMIR is to coat the surface of isolated islets with inhibitory molecules, thereby locally inhibiting the coagulation and complement systems at the transplant site. A 14-patient Phase 1/2a study in New Zealand showed that neonatal porcine islets encapsulated with alginate-poly-L-ornithine-alginate (APA) were safe and reduced unawareness of hypoglycemia in patients with type 1 diabetes (221). Strategies such as donor-specific hematopoietic progenitor cell transplantation (mixed chimerism) and concomitant donor-specific thymus transplantation showed great promise for improving immune tolerance (221, 222).

### 4.2 Early acute rejection

Studies have shown that during early acute rejection of porcine islet xenografts, the rejecting host graft exhibits direct and indirect anti-donor T cell IL-17 responses and produces strong anti-pig antibodies with severe B cell infiltration (148). IL-17 produced by the early donor stimulus dominates the early acute rejection response rather than IFN-γ production. Treatment with porcine ECDI-SP inhibits the host anti-pig IL-17 response, and when combined with transient B cell depletion (such as anti-CD20 monoclonal antibody) and short-course sirolimus, this triple therapy significantly and durably suppresses the host anti-pig IL-17 response and significantly prolongs the survival time of porcine islet xenografts (223). During early acute rejection, B cells may help induce the differentiation of IL-17-producing T cells and the production of xenogeneic antibodies by plasma cells. Studies have shown that B cell antigens presented by B1 B cells can effectively promote Th17 differentiation (224–226). Conversely, Th17 cells are effective B cell helper cells that can induce B cell proliferation *in vitro* and trigger their class switching *in vivo (* 227). It can be imagined that the induced xenogeneic IL-17 response feeds back to promote B cell proliferation and differentiation, establishing a positive feedback loop between B cells and Th17 cells, effectively promoting early acute rejection of islet xenografts.

### 4.3 Late rejection

In the context of late rejection initially protected by porcine ECDI-SP + anti-CD20 + sirolimus triple therapy, it was found that late rejection appeared to be entirely cell-mediated, as xenogeneic antibodies could not be detected after the rejection of the islet xenografts. Secondly, the phenomenon of late rejection seemed to always be associated with highly aggressive B cell infiltration in the graft. Thirdly, indirect xenogeneic IFN-γ responses appeared before the late rejection after B cell reconstitution (148). It can be imagined that newly emerged B cells directly acquire xenogeneic antigens in the graft and induce indirect anti-donor IFN-γ responses. Graft-infiltrating B cells may also directly initiate cytotoxic T lymphocytes within the graft, leading to the *in situ* destruction of the graft (228).

## 5 Strategies for treating the immune response to xenogeneic islet transplantation

### 5.1 Islet encapsulation

Islet encapsulation is an advanced method of islet transplantation, where isolated islets from humans or pigs can be transplanted without the need for toxic immunosuppression. This proves particularly beneficial for porcine islet xenotransplantation. Encapsulating islets with a semipermeable barrier allows for the exchange of nutrients and hormones, including insulin, while maintaining immune isolation, thus overcoming one of the major obstacles of xenotransplantation. Although clinical trials of porcine islets have achieved some success in New Zealand and Argentina, more research may be needed to develop optimal encapsulation methods and materials before this technology is ready for larger clinical trials in the United States. Key factors influencing encapsulation technology include:

**1. Capsule size and material:** Traditionally, smaller capsules are believed to be more effective due to easier material exchange through the capsule (229). However, recent studies suggest that spherical materials with diameters ≥1.5 mm exhibit significantly better biocompatibility compared to smaller or differently shaped counterparts (230). An *in vivo* study demonstrated that 1.5 mm alginate-encapsulated rat islets could restore blood glucose control in streptozotocin-induced diabetic C57 BL/6 mice for up to 180 days. This indicates that biocompatibility might be more crucial than material exchange efficiency in terms of effectiveness. Alginate consists of linear binary copolymers of β-D-mannuronic acid and α-L-guluronic acid. The length and sequence of mannuronic and guluronic acid chains in alginate hydrogels, as well as the mannuronic to guluronic acid ratio (M

ratio), determine alginate’s mechanical strength, elasticity, durability, permeability, and swelling properties. The use of multivalent cations (Ca²^+^, Ba²^+^) and polycations (poly-L-lysine or poly-L-ornithine) during alginate synthesis alters its properties (231). For example, alginate-poly-L-ornithine capsules provide high biocompatibility, better stability, and improved mechanical strength but induce excessive pericapsular cell overgrowth and macrophage activation, leading to capsule fibrosis. Multivalent cations like barium can avoid such fibrosis but reduce molar selectivity.

**2. Transplantation site:** Since the early 1970s, the liver infused via the portal vein has been widely accepted as the optimal site for islet transplantation in rodents. This principle, due to the prevalence and importance of rodent studies, has been extended to most animal models and nearly become the preferred site for microencapsulated islet transplantation (232). However, subsequent studies have identified several reasons why the liver is not the best site, including: (1) interaction with blood flow causing IBMIR, reducing islet mass by up to 50%; (2) the possibility of thrombosis during infusion; (3) relatively lower oxygen tension compared to the pancreas (233, 234). Intraperitoneal transplantation is also common, mainly due to its low volume limitation on grafts. However, this site has several drawbacks, including lack of close contact with blood flow, uncertain distribution of encapsulated islets, and the tendency for capsules to stack in the pelvic cavity of bipedal animals, making them difficult to retrieve. Subcapsular kidney transplantation is often used in animal models, considering the large number of encapsulated islets used for clinical transplantation and the lack of aggregation at this site. The subcutaneous space can be used for large numbers of encapsulated islets; however, this site is notoriously poor for blood access. Prevascularized subcutaneous spaces seem promising for both device and device-free methods (235), though this area requires two surgeries for prevascularization and actual transplantation. The omental pouch can also be used without two surgeries, potentially making it an ideal site for encapsulated islet implantation. In fact, studies using immunocompetent diabetic rat models have shown long-term function of encapsulated islets in the omental pouch (236). Researchers have also explored potential sites such as the gastric submucosa, peritoneal space, spleen, bone, and muscle. Animal models have identified specific advantages of several alternative sites, such as low blood contact to reduce IBMIR or the ability to biopsy the site after islet delivery, but to date, these positive results have been offset by equally compelling negative factors such as insufficient oxygen supply, surgical difficulty, or the need for more islets to correct blood glucose imbalance. Ongoing research may eventually yield a better site for islet infusion, though the liver remains the best choice for clinical islet transplantation despite its recognized limitations.

Porcine and microencapsulated islets have both been used clinically without significant side effects. However, compared to allogeneic naked islet transplantation, this method’s effectiveness is still suboptimal. Improving islet quality, enhancing capsule biocompatibility, and determining suitable implantation sites are crucial for the implementation of this therapy (237). Further research should make this method as effective as allogeneic naked islet transplantation, representing a real breakthrough in overcoming donor shortages and avoiding or mitigating the side effects associated with immunosuppressive drugs.

### 5.2 Immunosuppressants for xenotransplantation

In 1994, CG Groth reported the first xenotransplantation of porcine fetal islet-like cell clusters in a T1D patient. This study demonstrated the feasibility of porcine islet transplantation but did not show improvement in the patient’s condition (238). Over the following decades, porcine islet xenotransplantation has been more thoroughly explored in preclinical trials with non-human primates (239, 240). Humoral rejection is the main obstacle to the success of xenotransplantation. The α1,3Gal epitope, present on the surface of almost all animals except humans and some primates, is the primary antigen causing hyperacute rejection in pig-to-human and pig-to-non-human primate islet xenotransplantation. The University of Pittsburgh and Revivicor, Inc. designed Gal knockout (GTKO) pigs that do not express Gal (158). This proved to be a significant milestone in the development of xenotransplantation. Although other xenogeneic antigens were later discovered, Gal remains the most relevant, and GTKO pigs are considered a potential choice for eventual clinical translation. However, Gal knockout does not prevent islet rejection, and other genetic manipulations have been explored. In 2009, the Pittsburgh islet team first demonstrated long-term function (up to one year) of islet grafts in streptozotocin-induced diabetic non-human primates transplanted with porcine islets genetically modified to express human complement regulatory protein (hCD46). hCD46 expressed on porcine islets limited antibody-mediated rejection, allowing for the reduction of immunosuppression to maintain sufficient islet mass for long-term normal function. However, it did not reduce the initial islet loss associated with IBMIR as expected (241). This led to the further development of multigene pig islet donors capable of providing multifaceted protection to enhance islet transplantation. Five years later, the same group achieved similar success, long-term transplantation of islets from multigene pigs for the first time. A pig with four modified genes, (i) GTKO, and (ii) hCD46, (iii) human tissue factor pathway inhibitor (hTFPI) for antithrombotic and anti-inflammatory effects, and (iv) CTLA4-Ig to inhibit cellular immune responses, demonstrated improved success rates in retaining islet mass early postoperatively and maintaining islet implantation and function for up to one year during transplantation (242). This study also provided preliminary insights into glucose metabolism in pigs expressing human genes regulated by the insulin promoter, demonstrating that multiple islet-targeting transgenes inserted into pigs were not harmful to islet function and opened the door to further experiments and genetic manipulation for islet xenotransplantation (243). Multigene donor pigs have been shown to be a reproducibly effective source of islets for pig-to-non-human primate xenotransplantation (209). CG Park and colleagues at Seoul National University in Korea are conducting ongoing research of great significance for islet xenotransplantation, successfully maintaining normal blood glucose levels in diabetic primates within 600 days post porcine islet transplantation (244). A common feature of these successful long-term porcine-to-non-human primate islet studies is the use of CD154 monoclonal antibody (mAb)-based immunosuppression to prevent rejection. Although there is evidence that anti-CD154 mAb is effective and safe in pig-to-non-human primate islet transplantation models (245), it is associated with thromboembolic complications in humans and is not clinically translatable.

Despite promising data on the use of anti-CD40 antibody (co-stimulatory blockade) in organ xenotransplantation (246, 247), the islet xenotransplantation community is still searching for a clinically translatable immunosuppressant that can successfully prevent rejection without causing excessive side effects. New techniques for targeted genomic editing, particularly clustered regularly interspaced short palindromic repeats (CRISPR)-associated protein-9 nuclease (Cas9), offer hope that further genetic manipulation of porcine islets can improve compatibility between host and donor, thus allowing successful control of rejection with previously unfeasible immunosuppression. The field of islet xenotransplantation is steadily advancing and may soon approach clinical-grade experience and technology to begin clinical trials (248).

## 6 Conclusion and perspectives

### 6.1 Affirming islet transplantation

Islet transplantation holds great promise for the treatment of T1DM, as it offers the potential to restore euglycemia in a reliable manner, protects against hypoglycemia and glycemic liability in a way that exogenous insulin administration has thus far been unable to achieve. It has reduced many complications of diabetes and greatly improved patient healing.

### 6.2 Role and limitations of immunosuppressants

Limited islet survival after implantation hinders the success of IT due to innate immune attack through IBMIR, recurrent autoimmune islet destruction, or alloimmune rejection. The need for lifelong immunosuppressive therapy and the attendant risks of infection, cancer, and nephrotoxicity pose their own unique additional challenges, making this treatment unattractive to all but those at risk of severe brittle hypoglycemia. Optimizing new blood vessel formation by better controlling angiogenesis, suppressing inflammation, and reducing oxidative stress can all further improve outcomes.

### 6.3 Challenges and difficulties

The number of islets available for transplantation is a major limitation for both autoislet and alloislet approaches to β-cell replacement therapy. Therefore, the establishment of an unlimited source of islet tissue for transplantation has been a long-sought-after goal.

### 6.4 Future research directions

**Stem cells:** Significant progress has been made in the science and application of pluripotent stem cells, which are now entering early-stage pilot clinical trials. The possibility that cell transplantation can be accomplished with less need for immunosuppressants remains a real possibility, and progress is being made in immunomodulatory control through Treg infusion, MSC co-transplantation, and other innovative approaches.

**Porcine islet xenotransplantation:** Porcine islets have the advantage of targeting normal insulin similar to that present in humans, as well as the physiological ability to handle the heavy demands of insulin secretion. Importantly, porcine IAPP contains amino acid substitutions in the region corresponding to residues 20 to 29 that prevent the formation of fibrils (249, 250). Disadvantages include the larger immunologic barrier of xenogeneic than allogeneic tissue that presents an additional risk for hyperacute rejection and requires more intensive immunosuppression (239, 240),

**Islet encapsulation technology:** Islet encapsulation provides a barrier to protect transplanted islets, mainly by preventing excessive fibrosis, promoting local vascularization, and preventing future chronic immunosuppressive rejection. The latest data from NOD mice appear to confirm that agarose microencapsulated islets protect against autoimmune reactions (251). A recent paper published in PNAS shows that large encapsulated islets placed in the omentum protect grafts from immune attack and improve glucose metabolic control (252). These data demonstrate the potential of this technique as a safe method for successful islet transplantation.
